# P20 Treatment resistant psoriatic arthritis: a case report

**DOI:** 10.1093/rap/rkac067.020

**Published:** 2022-09-28

**Authors:** Arani Vivekanantham, Mihaela Florea, Laura Coates

**Affiliations:** University of Oxford, Oxford, United Kingdom; Oxford University Hospitals, Oxford, United Kingdom; Dorset County Hospital, Dorset, United Kingdom; University of Oxford, Oxford, United Kingdom; Oxford University Hospitals, Oxford, United Kingdom

## Abstract

**Introduction/Background:**

Although the era of targeted drugs has transformed the treatment of psoriatic arthritis (PsA), treatment resistant PsA still occurs. We present a rare case of a patient (renamed Ms Smith) with severe treatment resistant PsA.

Ms Smith, a previously healthy 25-year-old, was diagnosed with arthritis in July 2018. She presented at that time with severe monoarthritis of right ankle starting in September 2017. It was initially thought to be possible sarcoma, but biopsy confirmed inflammation. She had no psoriasis and was HLA-B27 positive. She was diagnosed seronegative peripheral spondyloarthritis (psoriatic type).

**Description/Method:**

She was treated with sulfasalazine and methotrexate in September 2018 but had side effects and no response. Her arthritis spread to multiple joints (tender joint count (TJC) 8, swollen joint count (SJC) 5, patient/physician global 4) and treatment was escalated to adalimumab biosimilar, but with minimal improvement (6 TJC, 5 SJC, patient/physican global 4). Despite additional prednisolone and subcutaneous methotrexate, she did not make a significant response.

Given her poor response, she was switched to secukinumab in March 2019 along with short course prednisolone. At 12 weeks she had partial response from physician assessment, but she did not feel much better. To maximise response, she was also given leflunomide but experienced side effects and no benefit. She also developed bloody diarrhoea, raising concern about inflammatory bowel disease (a recognised complication of IL-17), but investigations were negative. Given the side-effects and non-response, secukinumab and leflunomide were stopped after 6 months.

In January 2020, she presented with TJC 24, SJC 6 and patient/physician global of 5. She was switched to tofacitinib and restarted on leflunomide with subcutaneous methotrexate. Again, she stopped leflunomide but continued tofacitinib plus methotrexate until August 2020. At that time, she had presented with left hip pain; MRI confirmed sacroiliitis and left hip synovitis. She also had multiple swollen joints. Given worsening disease and spinal involvement, she was switched to etanercept as fourth biologic but did not respond to this either.

**Discussion/Results:**

She saw a second rheumatologist who agreed with diagnosis/treatments. Bloods tests, illustrated in Table 1, showed increase in her CRP. X-rays reviewed by radiology specialist interestingly showed significant erosive disease as well as unexpectedly some subluxation that appeared more like RA than PsA. Following an IFR, she was started on tocilizumab (subcutaneously initially and then one intravenously) but developed a non-allergic reaction to it (so only given half the infusion) and unusually her CRP increased to 325.

A PET-CT scan showed FDG avid nodes in the axillae, abdominal retroperitoneum, pelvis, and inguinal regions. A lymph node biopsy found IgG4 cells and reactive changes to a tattoo. Discussion in regional IgG4 meeting felt IgG4 disease was unlikely. Echocardiogram and blood cultures were negative.

She was recently started on rituximab, alongside methotrexate. If no response to this - what next? Cyclophosphamide? She has recently been diagnosed with Human Papillomavirus and had Cervical Intraepithelial Neoplasia 1 on colposcopy.

**Key learning points/Conclusion:**

Ms Smith has one of the most resistant forms of arthritis that did not respond to any treatment to date.

**P20 Table 1:** Blood test results

Blood tests	Results
C-reactive protein	127 (previously consistently around 80-90)
Rheumatoid factor	Negative
Anti-cyclic citrullinated peptides (anti-CCP)	Negative
Anti-nuclear antibodies	Negative
Proteinase 3 (PR3)	Normal (2.5)
Myeloperoxidase (MPO)	Borderline positive (4.3)
IgG4	Very raised (no antibodies identified)
Triglycerides	Normal
Ferritin	300

